# An Assessment of the Methodological Quality of Published Network Meta-Analyses: A Systematic Review

**DOI:** 10.1371/journal.pone.0121715

**Published:** 2015-04-29

**Authors:** James D. Chambers, Huseyin Naci, Olivier J. Wouters, Junhee Pyo, Shalak Gunjal, Ian R. Kennedy, Mark G. Hoey, Aaron Winn, Peter J. Neumann

**Affiliations:** 1 Center for the Evaluation of Value and Risk in Health, Institute for Clinical Research and Health Policy Studies, Tufts Medical Center, 800 Washington Street, #63, Boston, Massachusetts, 02111, United States of America; 2 LSE Health and Social Care, Cowdray House, London School of Economics and Political Science Houghton Street, London, WC2A 2AE, United Kingdom; 3 WHO Collaborating Centre for Pharmaceutical Science and Regulation, Division of Pharmacoepidemiology and Clinical Pharmacology, Utrecht University, P.O. Box 80082, 3508 TB, Utrecht, The Netherlands; 4 Precision Health Economics, 9433 Bee Caves Road, Suite 255B, Austin, Texas, 78733, United States of America; 5 Royal Victoria Hospital, 274 Grosvenor Road, Belfast, BT12 6BA, United Kingdom; 6 Mater Hospital, 45–54 Crumlin Road, Belfast, BT14 6AB, United Kingdom; 7 Department of Health Policy and Management, The University of North Carolina at Chapel Hill, 135 Dauer Drive, 1101 McGavran-Greenberg Hall, CB #7411, Chapel Hill, North Carolina, 27599, United States of America; Heinrich-Heine-University and University Hospital Duesseldorf, GERMANY

## Abstract

**Objective:**

To assess the methodological quality of published network meta-analysis.

**Design:**

Systematic review.

**Methods:**

We searched the medical literature for network meta-analyses of pharmaceuticals. We assessed general study characteristics, study transparency and reproducibility, methodological approach, and reporting of findings. We compared studies published in journals with lower impact factors with those published in journals with higher impact factors, studies published prior to January 1^st^, 2013 with those published after that date, and studies supported financially by industry with those supported by non-profit institutions or that received no support.

**Results:**

The systematic literature search identified 854 citations. Three hundred and eighteen studies met our inclusion criteria. The number of network meta-analyses has grown rapidly, with 48% of studies published since January 2013. The majority of network meta-analyses were supported by a non-profit institution or received no support (68%). We found considerable inconsistencies among reviewed studies. Eighty percent reported search terms, 61% a network diagram, 65% sufficient data to replicate the analysis, and 90% the characteristics of included trials. Seventy percent performed a risk of bias assessment of included trials, 40% an assessment of model fit, and 56% a sensitivity analysis. Among studies with a closed loop, 69% examined the consistency of direct and indirect evidence. Sixty-four percent of studies presented the full matrix of head-to-head treatment comparisons. For Bayesian studies, 41% reported the probability that each treatment was best, 31% reported treatment ranking, and 16% included the model code or referenced publicly-available code. Network meta-analyses published in higher impact factors journals and those that did not receive industry support performed better across the assessment criteria. We found few differences between older and newer studies.

**Conclusions:**

There is substantial variation in the network meta-analysis literature. Consensus among guidelines is needed improve the methodological quality, transparency, and consistency of study conduct and reporting.

## Introduction

Network meta-analysis (NMA) is increasingly accepted as a valuable methodology by clinical researchers and health care decision makers.[1–3] Network meta-analysis has been described as the “new generation” of evidence synthesis and the number of published studies is growing rapidly.[4] The approach has been embraced by national health technology assessment agencies in a number of countries, including Australia, Canada, and the UK.[5–7] The Cochrane Collaboration has also introduced a new type of review called ‘Overviews of Reviews’, which summarize the relative effectiveness of multiple competing interventions for a single indication.[8,9] A report by the Agency for Healthcare Research and Quality in the US identified 25 publicly-available guidance documents on how to conduct and report network meta-analyses.[10] National and regional health technology assessment agencies prepared the majority of these documents, although the guidance issued by the International Society for Pharmacoeconomics and Outcomes Research (ISPOR) was considered to be most comprehensive.[10]

The objective of this study was to assess the methodological quality of published NMAs. Previous studies have documented the growth and study characteristics of the NMA literature through July 2012.[3,4,11–14] These reviews investigated the validity assumptions and methods of using NMAs. Our study builds on this body of literature in several ways. First, we update the evidence by including articles published up to July 2014. Second, we investigate the methods, the transparency and reproducibility, and the presentation of findings in these studies. Finally, we compare the characteristics of studies published in journals with lower impact factors with those published in journals with higher impact factors, studies published prior to January 1^st^, 2013 with those published after that date, and studies supported financially by industry with those supported by non-profit or that received no support.

## Methods

We systematically searched for all published network meta-analyses in the Ovid-MEDLINE database using the following search terms: *network meta-analysis; indirect treatment comparison; mixed treatment comparison*; and, *multiple treatments meta-analysis*. The date of our last search was July 30^th^, 2014. We limited our search to studies including randomized controlled trials with human participants; we only included articles published in English-language journals. We excluded NMAs submitted to national health technology assessment agencies unless they were subsequently published in the medical literature.

Two trained reviewers reviewed each abstract independently using predefined inclusion criteria. We only included NMAs with at least one pharmaceutical in the set of treatments examined and data from at least three clinical trials; we also only included NMAs that compared the efficacy of the treatments. We excluded methodological studies, cost-effectiveness studies, editorials, and letters to the editor. Two trained reviewers independently extracted data from each included study; the reviewers resolved disagreements through consensus. We reviewed all published material related to each NMA, including the online supplementary material and appendices. When the source of study funding was undeclared or unclear, we contacted the corresponding author for the information.

Using a standardized data collection form, we collected information for each NMA on general study characteristics, study methods, transparency, and presentation of findings. We reported the following study characteristics: journal name; study authors; publication year; study support (industry or other); journal impact factor (as reported on Journal Citation Reports)[15]; country (determined by the corresponding author’s affiliation); number of included active treatments; number of clinical trials included; total number of patients included; and indication and patient population studied. We also noted all of the comparator treatments, including non-pharmaceutical treatments and different modes of administration of the same pharmaceutical.

We applied objective assessment criteria from the “checklist of good research practices” in the ISPOR guidance for interpreting and conducting indirect-treatment-comparison and network-meta-analysis studies.[16,17] Our assessment criteria included the following:

Study method assessment criteria
  - Was a Bayesian or a frequentist framework used?
  - Was the risk of bias of included clinical trials assessed? (e.g., using the Cochrane Collaboration's tool for assessing risk of bias or the Jadad scale?) [18,19]
  - Did the analysis include adjustments for model covariates?
  - Was a fixed or random effects model used? Or, were the findings of both fixed and random effects models presented?
  - Was an assessment of model fit reported?
  - Was a sensitivity analysis performed? (e.g., varied the included clinical studies to evaluate the robustness of the findings)
  - For studies with at least one closed loop, was the consistency of direct evidence and indirect evidence evaluated? (i.e., presented and compared the findings from the traditional meta-analysis and the network meta-analysis)
Study transparency and reproducibility assessment criteria
  - Were the search terms reported?
  - Was a network diagram of included treatments presented?
  - Was data from the included clinical studies necessary to reproduce the network meta-analysis presented?
  - Was a table of key clinical study characteristics presented?
  - Was the model code presented or source cited? (reported for studies performed using a Bayesian framework only)
Presentation of study findings
  - Were pairwise comparisons of all included treatments presented?
  - Was the probability of each treatment being best reported? (reported for studies performed using a Bayesian framework only)
  - Was a ranking of treatments in terms of effectiveness reported? (reported for studies performed using a Bayesian framework only)

To compare studies published in high- and low-impact journals, we evenly split the sample based on the reported journal impact factors (cut-off point: impact factor above and below 3.534). To compare older and newer studies, we again divided the sample in two (cut-off point: published before and after January 1^st^, 2013). We used our categorization of study support to compare industry-supported studies with studies with and non-industry-supported studies. We used a Chi-square test and a student’s t-test to evaluate categorical and continuous variables, respectively. We considered p-values below the 5% level as statistically significant. Analyses were undertaken using Stata 13.

## Results

**Fig 1:**
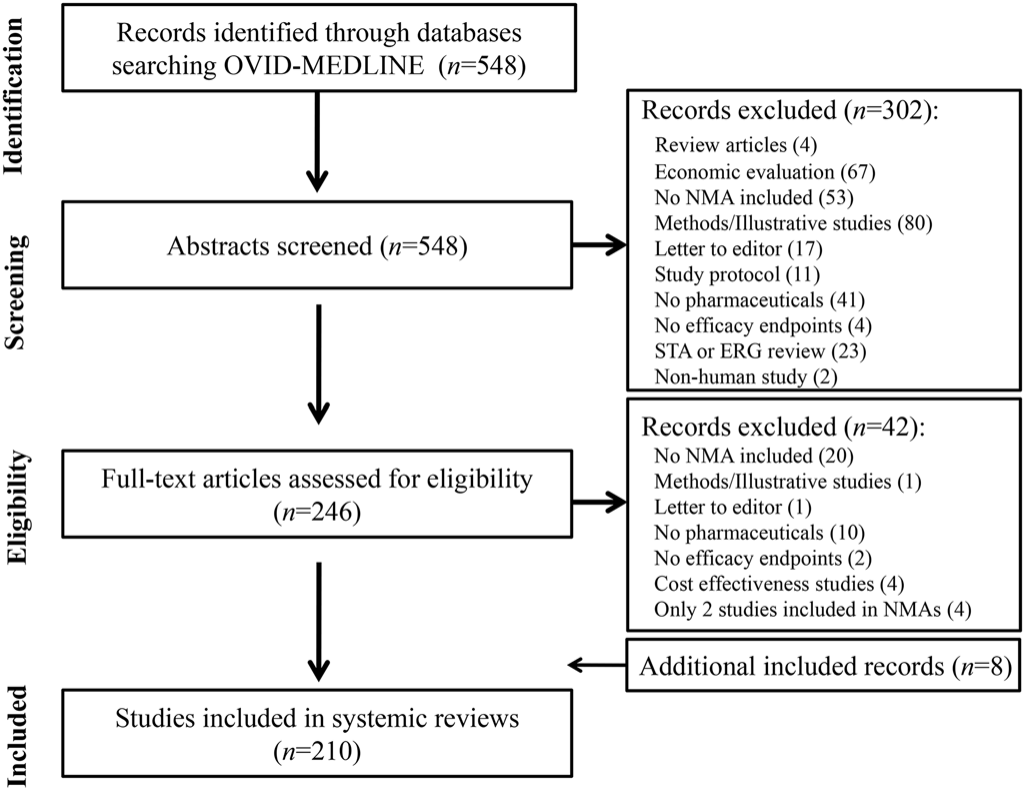
Identification of network meta-analyses included in review.

Our systematic literature search identified 854 citations. We reviewed the full texts of 386 abstracts met the inclusion criteria. Based on the full text reviews, we excluded another 68 studies. We included 318 studies in our final sample (Fig 1).

Table 1 presents the frequency of published NMAs by year, indication, and country; it also presents the types of included pharmaceutical included. The number of NMAs has increased rapidly each year; about 68% and 48% of studies were published after January 2012 and January 2013, respectively. Studies have evaluated treatments for a range of indications, most commonly those affecting the circulatory (n = 64, 20%) and musculoskeletal systems (n = 45, 14%). Analyses were most often performed by researchers based in the USA (n = 81, 26%), UK (n = 79, 25%), and Canada (n = 28, 9%). The majority of studies adopted a Bayesian framework (n = 214, 67%) and either received non-profit or no support (n = 215, 68%).

**Table 1:** Frequency of network meta-analyses (n = 210) by year, indication, and country.

Year study published^†^	n
1997	1 (0.3%)
2003	3 (0.9%)
2004	1 (0.3%)
2006	3 (0.9%)
2007	3 (0.9%)
2008	9 (2.8%)
2009	16 (5.0%)
2010	21 (6.9%)
2011	44 (13.8%)
2012	66 (20.4%)
2013	43 (24.5%)
2014 (through July 31st)	73 (23.0%)
**International Statistical Classification of Diseases (ICD) disease categories**	**n**
Blood Disease	3 (0.9%)
Circulatory System	64 (20.1%)
Digestive System	13 (4.1%)
Endocrine, Nutritional, Metabolic, and Immunity	28 (8.8%)
Genitourinary System	7 (2.2%)
Infectious and Parasite Disease	14 (4.4%)
Mental and Behavioral Disorder	13 (4.1%)
Musculoskeletal System and Connective Tissue	45 (14.2%)
Neoplasm	39 (12.3%)
Nervous System and Sensory Organs	33 (10.4%)
Respiratory System	20 (6.3%)
Skin and Subcutaneous Tissues	9 (2.8%)
Other	30 (9.4%)
**Country**	**n**
USA	81 (25.5%)
UK	79 (24.8%)
Canada	28 (8.8%)
Italy	21 (6.6%)
China	16 (5.0%)
France	14 (4.4%)
The Netherlands	10 (3.1%)
Germany	8 (2.5%)
Brazil	6 (1.9%)
Switzerland	6 (1.9%)
Taiwan	6 (1.9%)
Greece	5 (1.6%)
Spain	4 (1.3%)
Other	34 (10.7%)
**Type of pharmaceutical intervention included**	**n**
Multiple pharmaceuticals compared	304 (95.6%)
Study included a non pharmaceutical treatment (e.g., surgery, exercise, counselling, etc)	30 (9.4%)
Different strengths of the same pharmaceutical compared (e.g., simvastatin 20mg vs. 40mg)	82 (25.8%)
Treatments in the same drug class grouped together as a comparator (e.g., beta-blockers, or statins)	75 (23.6%)
Multiple modes of administration of a drug compared (e.g., oral, sublingual, intramuscular, etc)	10 (3.1%)

^†^ We limited our literature search to studies published in the medical literature. We did not include NMAs submitted to national health technology assessment agencies unless also published in the Ovid-MEDLINE database.

* ‘Other countries’ includes Greece, Ireland, Singapore, Australia, Cameroon, Denmark, Finland, Hong Kong, Korea, Norway, Poland, and Portugal.

The vast majority of network meta-analyses compared multiple pharmaceuticals (n = 304, 96%), while a minority included a non-pharmaceutical comparator treatment (n = 30, 9%). Some researchers grouped treatments in the same therapeutic class, e.g., beta-blockers or statins, as a single comparator treatment (n = 75, 24%). Different dosages, e.g., simvastatin 20 mg vs. 40 mg, were compared in 82 (26%) studies, and ten studies (3%) compared different modes of administration of the same pharmaceutical, e.g., oral vs. intramuscular injection.

We found many differences among the included studies (Table 2). In terms of study methods, 30% of studies did not perform a risk of bias assessment of included studies, 60% did not report an assessment of model fit, and 44% did not include a sensitivity analysis. Among studies with a closed loop, i.e., three or more included treatments had been compared in head-to-head trials, 40% did not report the consistency of direct and indirect evidence. In terms of study transparency and reproducibility, 20% did not report the systematic review search terms, 39% did not present a network diagram, 35% did not present sufficient data from contributing studies to replicate the analysis, and 10% did not provide details of the key characteristics of included trials. Thirty-six percent of included studies did not present the full matrix of head-to-head treatment comparisons. For studies performed using a Bayesian framework, 41% reported the probability that each included treatment was best, 31% reported a ranking of included treatments, and 16% included or directed readers to the model code.

**Table 2:** Assessment of network meta-analysis study characteristics.

		Journal quality (n = 301)*			Date of study publication (n = 318)			Source of study support (n = 315)**		
Assessment criteria	All studies (n = 318)	Low impact factor (<3.534) (n = 147)	High impact factor (≥3.534) (n = 154)	p-value	Older studies (published prior to 2013) (n = 167)	Recent studies (2013, 2014) (n = 151)	p-value	Industry support (n = 98)	Non-Industry support/ no support (n = 217)	p-value
***General study characteristics***										
Number of treatments compared	6.3 (±6.4)	6.8 (±8.5)	6.0 (±3.9)	0.3136	6.0 (±4.2)	6.7 (±8.2)	0.3816	5.9 (±3.6)	6.5 (±7.3)	0.446
Total number of studies	32.9 (±45.5)	28.3 (±38.6)	36.5 (±46.9)	0.0992	30.5 (±50.2)	35.5 (±50.2)	0.3341	22.7 (±29.4)	37.4 (±50.5)	**0.0079**
Total number of patients	26875 (±65936)	21938 (±46061)	33292 (±82859)	0.1549	23711 (±49899)	30460 (±80375)	0.3732	10945 (±13183)	33864 (±77635)	**0.005**
HTA region (UK, AUS and Canada)^†^	110 (35%)	50 (34%)	56 (36%)	0.6709	68 (47%)	42 (28%)	**0.0156**	48 (49%)	62 (28%)	**0.0003**
Journal impact factor	5.5 (±6.2)	NA	NA	NA	5.8 (±6.5)	5.2 (±5.9)	0.3791	3.1 (±1.7)	6.5 (±7.1)	**<0.0001**
***Study method***										
Bayesian framework	214 (67%)	91 (62%)	109 (71%)	0.1038	106 (63%)	108 (72%)	0.1273	75 (77%)	139 (63%)	**0.0191**
Risk of bias assessment of included studies	223 (70%)	100 (68%)	111 (72%)	0.4446	103 (62%)	120 (79%)	**0.0005**	53 (54%)	170 (77%)	**<0.0001**
Adjustment for covariates	92 (29%)	35 (24%)	51 (33%)	0.0744	54 (32%)	38 (25%)	0.1601	37 (38%)	55 (25%)	**0.0205**
Random effects model***	221 (70%)	98 (67%)	114 (75%)	0.1609	116 (69%)	106 (71%)	0.7453	67 (68%)	155 (71%)	0.6243
Assessment of model fit	127 (40%)	53 (36%)	70 (45%)	0.0979	69 (41%)	58 (38%)	0.5985	46 (47%)	81 (37%)	0.0894
Sensitivity analysis	179 (56%)	73 (50%)	96 (62%)	**0.0267**	88 (53%)	91 (60%)	0.1752	57 (58%)	122 (58%)	0.6542
Consistency of direct and indirect evidence reported**** (closed loop studies only, n = 167)	116 (69%)	39 (57%)	73 (79%)	**0.0017**	57 (66%)	59 (73%)	0.3606	16 (39%)	100 (79%)	**<0.0001**
***Study transparency and reproducibility***										
Search terms reported	254 (80%)	112 (76%)	129 (84%)	0.1007	129 (77%)	125 (83%)	0.2201	61 (62%)	193 (88%)	**<0.0001**
Network diagram	194 (61%)	85 (58%)	101 (66%)	0.1671	103 (62%)	91 (60%)	0.7974	62 (63%)	132 (60%)	0.5829
Extracted data from contributing clinical studies	206 (65%)	87 (60%)	106 (69%)	0.0955	116 (69%)	91 (61%)	0.1011	58 (60%)	149 (68%)	0.1726
Table of key clinical study characteristics	286 (90%)	128 (87%)	141 (92%)	0.2084	145 (87%)	141 (93%)	0.0527	89 (91%)	197 (90%)	0.729
Model code (Bayesian framework only, n = 214)	35 (16%)	9 (6%)	24 (16%)	**0.0085**	24 (14%)	11 (7%)	**0.0439**	8 (8%)	27 (12%)	0.2811
***Presentation of study findings***										
Full matrix of head-to-head comparisons	203 (64%)	84 (57%)	108 (70%)	**0.0191**	110 (66%)	93 (62%)	0.4294	44 (45%)	159 (73%)	**<0.0001**
Reported probability of being best (Bayesian framework only, n = 214)	87 (41%)	32 (22%)	51 (33%)	**0.0277**	41 (25%)	46 (30%)	0.2389	25 (26%)	62 (28%)	0.623
Ranking of included treatments (Bayesian framework only, n = 214)	67 (31%)	26 (18%)	40 (26%)	0.0829	29 (17%)	39 (26%)	0.0664	11 (11%)	56 (26%)	**0.0031**

^†^ Regions in which submissions to HTA agencies generally require a NMA;

* 17 studies published in journals with no associated impact factor;

** 3 studies for which source of study support was unclear;

*** 77 studies reported both fixed and random effects models, 38 studies did not report models used;

**** Consistency only reported for studies with a closed loop

### Journal quality

We found that NMAs published in journals with higher impact factors more often performed a sensitivity analysis (62% versus 50%, p = 0.0267) and reported the full matrix of head-to-head comparisons (70% versus 57%, p = 0.0191). For studies with a closed loop, those published in journals with higher impact factors more often compared the consistency of direct and indirect evidence (79% versus 57%, p = 0.0017). For studies performed using a Bayesian framework, studies published in higher impact factor journals more often reported the probability of each treatment being best (33% versus 22%, p = 0.0277), and the model code (16% versus 6%, p = 0.0085).

### Publication date

We found that studies published prior to January 1^st^, 2013 were more often authored by researchers based in regions where HTA agencies include NMAs in their assessments (47% versus 28%, p = 0.0156). Studies published prior to January 1^st^, 2013 less often performed a risk of bias assessment of included trials (62% versus 79%, p = 0.005), but, for Bayesian studies, more often reported the model code or cited publicly-available code (14% versus 7%, p = 0.0439).

### Source of financial support

We found a number of differences between industry-supported studies and non-industry supported studies. Industry-supported studies included fewer trials (22.7 versus 37.4, p = 0.0079) and a smaller total number of patients (10945 versus 33864, p = 0.0050). A greater proportion of industry-funded studies were authored by researchers based in regions in which HTA agencies often include NMA in their assessments (49% versus 28%, p = 0.0079). Industry-supported studies were on average published in journals with lower impact factors (3.1 versus 6.5, p<0.0001).

Industry-supported studies more often used a Bayesian framework (77% versus 63%, p = 0.0191), and adjusted for study covariates (38% versus 25%, p = 0.0251); however, they less often performed a risk of bias assessment of included studies (54% versus 77%, p<0.0001), and, for Bayesian studies, less often compared the consistency of direct and indirect evidence (39% versus 79%, p<0.0001).

Industry-supported studies less often reported the search terms used in the systematic literature review (62% versus 88%, p<0.0001), and reported the full matrix of head-to-head comparisons (45% versus 73%, p<0.0001). For studies performed using a Bayesian framework, industry-supported studies less often reported a ranking of treatments (11% versus 26%, p = 0.0031).

## Discussion

Network meta-analyses are increasingly recognized as offering valuable information to the medical community. Growth in the NMA literature has been rapid, with roughly half of studies in our dataset published since January 1^st^, 2013. Our dataset includes studies by researchers based in 30 countries, confirming NMAs as a global research enterprise. We found notable variation across published network meta-analyses with respect to methods, transparency and reproducibility, and presentation of findings.

As expected, we found that studies published in higher-impact journals performed better across our assessment criteria, suggesting that higher-quality journals publish higher quality NMAs. Our findings may also point to a marginal improvement in the conduct and reporting of NMAs over time, with more recently published studies more often performing a risk of bias assessment of included trials. Interestingly, more recent Bayesian studies less often reported the model code, perhaps reflecting the fact that example model code has become easily accessible and freely available.[20] It is notable that a smaller proportion of studies are now authored by researchers based in regions where HTA agencies include network meta-analysis in their assessments. This likely reflects the widespread acceptance of NMA and the growing international use of the technique by researchers.

We also found differences among NMAs according to source of financial support. For instance, we found that industry-supported studies tended to be published in journals with a lower impact factor. Similarly, industry-supported studies were more often authored by researchers based in HTA regions, which is likely due to the publication of NMAs that were previously included in a submission to a regulatory agency. An interesting finding is that industry-sponsored studies less often used a Bayesian framework, the reason for which is unclear.

Consistent with the studies published in journals with higher impact factors, we found that industry-supported studies less often reported the full matrix of head-to-head comparisons. For NMAs with a closed loop, they less often compared the consistency of direct and indirect evidence. Like older analyses, industry-supported studies were less likely to perform a risk of bias assessment of included trials.

Other identified differences between industry-supported and non-industry-supported studies do not appear to be explained by journal quality or the data of publication. For instance, industry-supported studies included fewer contributing trials and total patients, and less often reported the systematic review search terms. Industry sponsored Bayesian network meta-analyses less often reported a ranking of included drugs based on efficacy, although they more often adjusted for covariates. However, as we emphasize below, these findings should be interpreted with caution due to limitations of our assessment criteria.

### Study limitations

While our assessment criteria were based on published guidelines for the conduct and reporting of network meta-analysis, they may have been insufficient to fully assess study quality. For example, while we determined whether a study reported a sensitivity analysis or assessed the model fit, we did not judge whether the approaches were appropriate or sufficient. We also did not evaluate whether the set of included treatments was appropriate or comprehensive. This is a salient consideration as the inappropriate inclusion or exclusion of treatments can significantly affect study findings.

Furthermore, a study may have not met an assessment criterion, or appear to have performed poorly on a given criterion, for good reason. For instance, while industry-supported studies included fewer contributing studies and total patients, this may reflect the precision of the study research question, rather than the how comprehensively the question was addressed. Similarly, while guidelines recommend assessing included trials for risk of bias, the influence of individual design components of individual randomized trials on the findings is variable. [21–23] While it is recommended that the consistency between direct and indirect evidence should be assessed, researchers may be more inclined to do so when there is apparent heterogeneity among the included trials.[24–28]

For indications for which the relative efficacy of existing treatments has been established by multiple NMAs, a researcher may choose to compare a newly-introduced treatment to existing interventions, rather than to report the full matrix. Moreover, it is reported that treatment rankings may at times be an unreliable metric for comparative efficacy and safety, and authors may choose not to present these findings.[29]

Our comparison of NMAs on the basis of study support has a number of additional limitations. First, while we took great care when categorizing studies as ‘industry-supported’ and contacted authors when source of support was ambiguous, we predominantly relied on the reported disclosures. Second, we did not compare the conclusions of industry-and non-industry-supported NMAs. To determine if industry-supported studies tend to produce more favourable conclusions about the study sponsors’ drugs, it would be necessary to identify and compare industry sponsored and non-industry sponsored trials that evaluate the same clinical research questions.[30,31] Unfortunately, while various NMAs have been conducted for some indications, these studies often considered different research questions and included different treatments. This prevented us from evaluating the NMA literature in this way.

### Moving forward

This research highlights the need for greater consistency in the conduct and reporting of NMAs. In addition to the ISPOR recommendations used here, there exist a multitude of guidelines and recommendations with notable differences. This suggests that a consensus, on which NMA methods are most appropriate has not been reached.[10] [32] While the development of guidelines advances the field, inconsistencies between guidelines may impede standardization. For example, while all guidelines are unanimous in stating the consistency between direct and indirect evidence should be assessed, there is disagreement with respect to the importance of testing model fit, the use of fixed- or random-effects models, and the exclusion or inclusion of covariates in the model.[10]

As NMAs are an extension of traditional meta-analyses, it seems logical that they should be held to similar methodological and reporting standards. For systematic reviews and traditional meta-analysis, the Preferred Reporting Items for Systematic Reviews and Meta-Analyses (PRISMA) protocol—which consists of a 27-item checklist and a four-phase flow diagram—has emerged as the standard reporting approach.[33] The forthcoming extension of the PRISMA guidelines for network meta-analysis may consolidate existing recommendations, and serve as the authoritative guideline.

Still, our study underscores that checklists should be considered only as guides to study quality. A NMA that satisfies all criteria on a given checklist may not necessarily be a high-quality study, and a study that does not meet various criteria may not necessarily be a low-quality study. Indeed, the recent ISPOR-AMCP-NPC Good Practice Task Force Report is a questionnaire, not a checklist, reflecting the challenge of creating a ‘one size fits all’ checklist for network meta-analyses.[34] It is important that users of network meta-analyses critique the research, and make their own judgment regarding study quality and accuracy. Journal editors should ensure that published network meta-analyses are transparent and reproducible, and that these studies present findings in an unambiguous and comprehensive manner.
