# Correction: An Assessment of the Methodological Quality of Published Network Meta-Analyses: A Systematic Review

**DOI:** 10.1371/journal.pone.0131953

**Published:** 2015-07-21

**Authors:** James D. Chambers, Huseyin Naci, Olivier J. Wouters, Junhee Pyo, Shalak Gunjal, Ian R. Kennedy, Mark G. Hoey, Aaron Winn, Peter J. Neumann

In the second paragraph of the Results the sentence describing the number of studies receiving non-profit or no support should read “The majority of studies adopted a Bayesian framework (n = 214, 67%) and either received non-profit or no support (n = 217, 69%).”

In the final paragraph of the Results the percentage of studies with a closed loop is incorrect. The correct sentence should read “Among studies with a closed loop, i.e., three or more included treatments had been compared in head-to-head trials, 31% did not report the consistency of direct and indirect evidence.”

Under Publication Date the p value for 62% versus 79% should read (62% versus 79%, p = 0.0005).

Under Source of Financial Support the p value for 49% versus 28% in the first paragraph should read (49% versus 28%, p = 0.0003).

Under Source of Financial Support the second paragraph should read “Industry-supported studies more often used a Bayesian framework (77% versus 63%, p = 0.0191), and adjusted for study covariates (38% versus 25%, p = 0.0205); however, they less often performed a risk of bias assessment of included studies (54% versus 77%, p∠0.0001), and, for closed loop studies, less often compared the consistency of direct and indirect evidence (39% versus 79%, p∠0.0001).”

In the Discussion the third paragraph should read “An interesting finding is that industry-sponsored studies more often used a Bayesian framework”

Fig 1 is incorrect in the published article. Please see the correct Fig 1 here.

**Fig 1:**
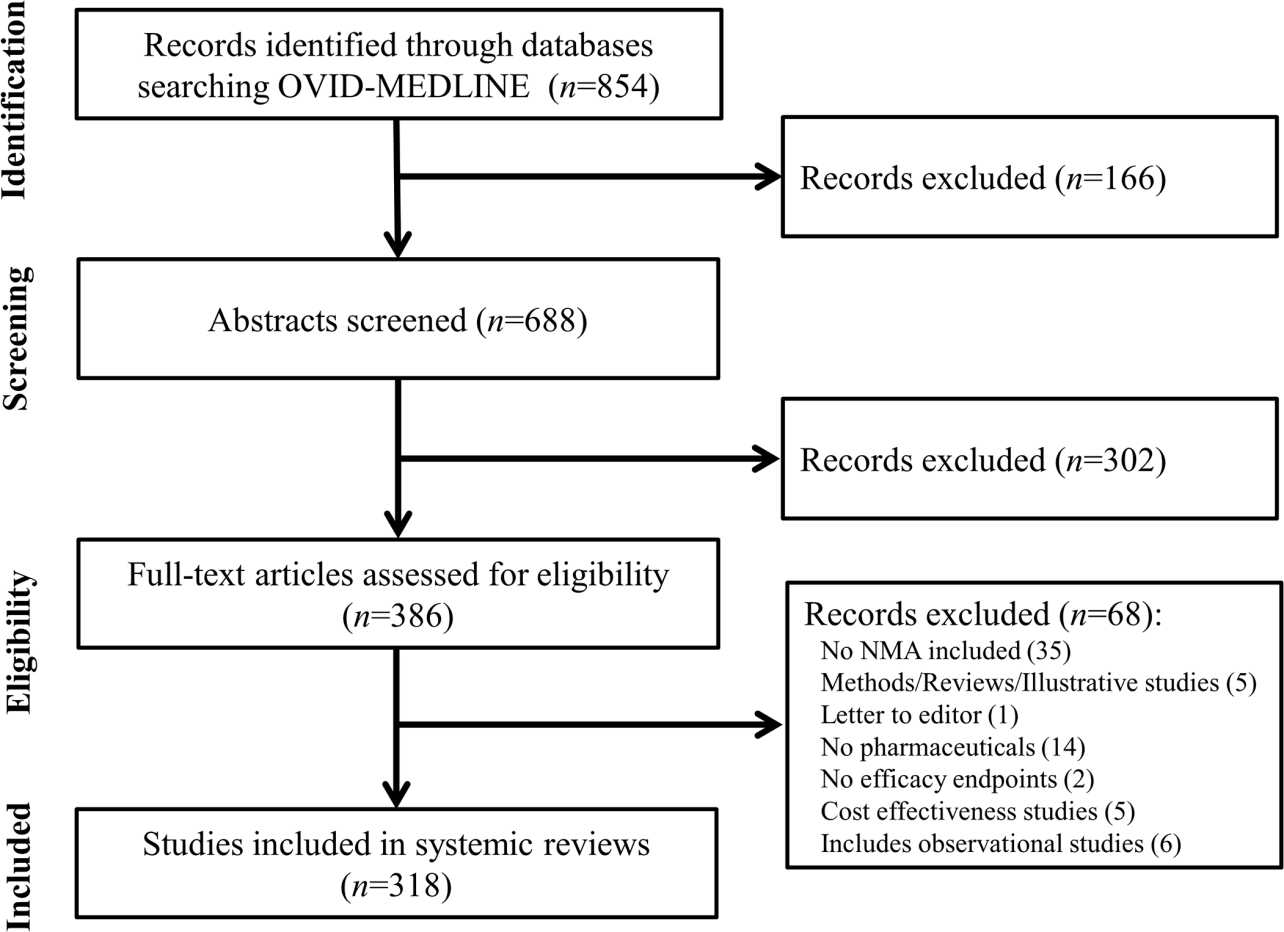
Identification of network meta-analyses included in review.

There are errors in Table 1 and Table 2 of the published article. Please see the correct tables here.

**Table 1:** Frequency of network meta-analyses (n = 318) by year, indication, and country

**Year study published†**	**n**
1997	1 (0.3%)
2003	3 (0.9%)
2004	1 (0.3%)
2006	3 (0.9%)
2007	3 (0.9%)
2008	9 (2.8%)
2009	16 (5.0%)
2010	21 (6.9%)
2011	44 (13.8%)
2012	66 (20.4%)
2013	78 (24.5%)
2014 (through July 31st)	73 (23.0%)
**International Statistical Classification of Diseases (ICD) disease categories**	**n**
Blood Disease	3 (0.9%)
Circulatory System	64 (20.1%)
Digestive System	13 (4.1%)
Endocrine, Nutritional, Metabolic, and Immunity	28 (8.8%)
Genitourinary System	7 (2.2%)
Infectious and Parasite Disease	14 (4.4%)
Mental and Behavioral Disorder	13 (4.1%)
Musculoskeletal System and Connective Tissue	45 (14.2%)
Neoplasm	39 (12.3%)
Nervous System and Sensory Organs	33 (10.4%)
Respiratory System	20 (6.3%)
Skin and Subcutaneous Tissues	9 (2.8%)
Other	30 (9.4%)
**Country**	**n**
USA	81 (25.5%)
UK	79 (24.8%)
Canada	28 (8.8%)
Italy	21 (6.6%)
China	16 (5.0%)
France	14 (4.4%)
The Netherlands	10 (3.1%)
Germany	8 (2.5%)
Brazil	6 (1.9%)
Switzerland	6 (1.9%)
Taiwan	6 (1.9%)
Greece	5 (1.6%)
Spain	4 (1.3%)
Other	34 (10.7%)
**Type of pharmaceutical intervention included**	**n**
Multiple pharmaceuticals compared	304 (95.6%)
Study included a non pharmaceutical treatment (e.g., surgery, exercise, counselling, etc)	30 (9.4%)
Different strengths of the same pharmaceutical compared (e.g., simvastatin 20mg vs. 40mg)	82 (25.8%)
Treatments in the same drug class grouped together as a comparator (e.g., beta-blockers, or statins)	75 (23.6%)
Multiple modes of administration of a drug compared (e.g., oral, sublingual, intramuscular, etc)	10 (3.1%)

† We limited our literature search to studies published in the medical literature. We did not include NMAs submitted to national health technology assessment agencies unless also published in the Ovid-MEDLINE database. * ‘Other countries’ includes Greece, Ireland, Singapore, Australia, Cameroon, Denmark, Finland, Hong Kong, Korea, Norway, Poland, and Portugal.

**Table 2:** Assessment of network meta-analysis study characteristics

Assessment criteria	All studies (n = 318)	Journal quality (n = 301)*			Date of study publication (n = 318)			Source of study support (n = 315)**		
		Low impact factor (<3.534) (n = 147)	High impact factor (≥3.534) (n = 154)	p-value	Older studies (published prior to 2013) (n = 167)	Recent studies (2013, 2014) (n = 151)	p-value	Industry support (n = 98)	Non-Industry support/ no support (n = 217)	p-value
***General study characteristics***										
Number of treatments compared	6.3 (±6.4)	6.8 (±8.5)	6.0 (±3.9)	0.3136	6.0 (±4.2)	6.7 (±8.2)	0.3816	5.9 (±3.6)	6.5 (±7.3)	0.446
Total number of studies	32.9 (±45.5)	28.3 (±38.6)	36.5 (±46.9)	0.0992	30.5 (±50.2)	35.5 (±50.2)	0.3341	22.7 (±29.4)	37.4 (±50.5)	**0.0079**
Total number of patients	26875 (±65936)	21938 (±46061)	33292 (±82859)	0.1549	23711 (±49899)	30460 (±80375)	0.3732	10945 (±13183)	33864 (±77635)	**0.005**
HTA region (UK, AUS and Canada)†	110 (35%)	50 (34%)	56 (36%)	0.6709	68 (41%)	42 (28%)	**0.0156**	48 (49%)	62 (28%)	**0.0003**
Journal impact factor	5.5 (±6.2)	NA	NA	NA	5.8 (±6.5)	5.2 (±5.9)	0.3791	3.1 (±1.7)	6.5 (±7.1)	**<0.0001**
***Study method***										
Bayesian framework	214 (67%)	91 (62%)	109 (71%)	0.1038	106 (63%)	108 (72%)	0.1273	75 (77%)	139 (63%)	**0.0191**
Risk of bias assessment of included studies	223 (70%)	100 (68%)	111 (72%)	0.4446	103 (62%)	120 (79%)	**0.0005**	53 (54%)	170 (77%)	**<0.0001**
Adjustment for covariates	92 (29%)	35 (24%)	51 (33%)	0.0744	54 (32%)	38 (25%)	0.1601	37 (38%)	55 (25%)	**0.0205**
Random effects model***	221 (70%)	98 (67%)	114 (75%)	0.1609	116 (69%)	106 (71%)	0.7453	67 (68%)	155 (71%)	0.6243
Assessment of model fit	127 (40%)	53 (36%)	70 (45%)	0.0979	69 (41%)	58 (38%)	0.5985	46 (47%)	81 (37%)	0.0894
Sensitivity analysis	179 (56%)	73 (50%)	96 (62%)	**0.0267**	88 (53%)	91 (60%)	0.1752	57 (58%)	122 (58%)	0.6542
Consistency of direct and indirect evidence reported**** (closed loop studies only, n = 167)	116 (69%)	39 (57%)	73 (79%)	**0.0017**	57 (66%)	59 (73%)	0.3606	16 (39%)	100 (79%)	**<0.0001**
***Study transparency and reproducibility***										
Search terms reported	254 (80%)	112 (76%)	129 (84%)	0.1007	129 (77%)	125 (83%)	0.2201	61 (62%)	193 (88%)	**<0.0001**
Network diagram	194 (61%)	85 (58%)	101 (66%)	0.1671	103 (62%)	91 (60%)	0.7974	62 (63%)	132 (60%)	0.5829
Extracted data from contributing clinical studies	206 (65%)	87 (60%)	106 (69%)	0.0955	116 (69%)	91 (60%)	0.1011	58 (60%)	149 (68%)	0.1726
Table of key clinical study characteristics	286 (90%)	128 (87%)	141 (92%)	0.2084	145 (87%)	141 (93%)	0.0527	89 (91%)	197 (90%)	0.729
Model code (Bayesian framework only, n = 214)	35 (16%)	9 (6%)	24 (16%)	**0.0085**	24 (14%)	11 (7%)	**0.0439**	8 (8%)	27 (12%)	0.2811
***Presentation of study findings***										
Full matrix of head-to-head comparisons	203 (64%)	84 (57%)	108 (70%)	**0.0191**	110 (66%)	93 (62%)	0.4294	44 (45%)	159 (73%)	**<0.0001**
Reported probability of being best (Bayesian framework only, n = 214)	87 (41%)	32 (22%)	51 (33%)	**0.0277**	41 (25%)	46 (30%)	0.2389	25 (26%)	62 (28%)	0.623
Ranking of included treatments (Bayesian framework only, n = 214)	67 (31%)	26 (18%)	40 (26%)	0.0829	29 (17%)	39 (26%)	0.0664	11 (11%)	56 (26%)	**0.0031**

† Regions in which submissions to HTA agencies generally require a NMA

* 17 studies published in journals with no associated impact factor

** 3 studies for which source of study support was unclear

*** 77 studies reported both fixed and random effects models, 38 studies did not report models used

**** Consistency only reported for studies with a closed loop
